# *Vitis* Flower Sex Specification Acts Downstream and Independently of the ABCDE Model Genes

**DOI:** 10.3389/fpls.2018.01029

**Published:** 2018-07-16

**Authors:** João L. Coito, Helena Silva, Miguel J. N. Ramos, Miguel Montez, Jorge Cunha, Sara Amâncio, Maria M. R. Costa, Margarida Rocheta

**Affiliations:** ^1^Linking Landscape, Environment, Agriculture and Food (LEAF), School of Agriculture, University of Lisbon, Lisbon, Portugal; ^2^Plant Functional Biology Centre, Biosystems and Integrative Sciences Institute, University of Minho, Braga, Portugal; ^3^Instituto Nacional de Investigação Agrária e Veterinária, Oeiras, Portugal

**Keywords:** *Vitis vinifera sylvestris*, *in situ* hybridization, homeotic genes, flower ABCDE model, development, dioecious

## Abstract

The most discriminating characteristic between the cultivated *Vitis vinifera* subsp. *vinifera* and the wild-form *Vitis vinifera* subsp. *sylvestris* is their sexual system. Flowers of cultivars are mainly hermaphroditic, whereas wild plants have female and male individuals whose flowers follow a hermaphroditic pattern during early stages of development and later develop non-functional reproductive organs. In angiosperms, the basic developmental system for floral organ identity is explained by the ABCDE model. This model postulates that regulatory gene functions work in a combinatorial way to confer organ identity in each whorl. In wild *Vitis* nothing is known about the function and expression profile of these genes. Here we show an overall view of the temporal and spatial expression pattern of the ABCDE genes as well as the pattern of *VviSUPERMAN* that establishes a boundary between the stamen and the carpel whorls, in the male, female and complete flower types. The results show a similar pattern in *Vitis* species suggesting that the pathway leading to unisexuality acts independently and/or downstream of B- and C- function genes.

## Introduction

Flower organ development is under the control of highly specialized genetic networks that have been well studied during the past decades (Wellmer and Riechmann, 2010; O’Maoileidigh et al., 2014). The genus *Vitis* presents a notorious variability of flower types. The flowers of the cultivated *V. v. vinifera* are mainly hermaphrodite, while the wild type, *V. v. sylvestris* is dioecious with male plants producing flowers with erect stamens but without pistils and female plants displaying flowers with a fully formed pistil but reflexed stamens with infertile pollen (Valleau, 1916; Carmona et al., 2008; Ramos et al., 2014). The shift in sexual system from dioecy to hermaphroditism in *Vitis* species is not yet completely understood.

Several attempts were made to understand and provide insight into the molecular mechanism regarding the origins of this sexual dimorphism present in *V. v. sylvestris* individuals. Several genetic mapping studies based on the 8x version of the *Vitis* genome^1^ annotation (Dalbó et al., 2000; Riaz et al., 2006; Marguerit et al., 2009) located a locus responsible for sex determination at the vicinity of the genetic markers VviMD34 and VviIB23 (Dalbó et al., 2000; Riaz et al., 2006) on chromosome 2 in the 8x version^2^. Using these markers putatively linked to the sex locus, a new genetic map was developed and refined to restrict the sex locus to 143 kb in the chromosome 2, between 4,907,434 and 5,050,616 bp (Fechter et al., 2012). A more recent study, focusing on the 143 kb region of chromosome 2 extended the sex locus region to 158 kb downstream of the genetic marker VviIB23 and encompassing the previous 143 kb region (Picq et al., 2014). This new locus showed haplotype diversity, linkage disequilibrium, and several genes segregating to typically associated X-Y sex determining region.

The canonical hermaphrodite flower structure can be divided into four whorls. First and second whorls comprise the sterile perianth of the flower, containing sepals and petals, respectively. Reproductive organs are formed in the innermost whorls, the stamens in the third whorl and carpel in the flower center, the fourth whorl (Dellaporta and Calderon-Urrea, 1993; Bowman et al., 2012). The first genes involved in flower organ identity were described in the model plants *Antirrhinum majus* and *Arabidopsis thaliana* (Coen and Meyerowitz, 1991). Functional analysis of these genes allowed the postulation of the ABC model that assumes that three classes of proteins act in a combinatorial way to confer organ identity in each whorl. In *A. thaliana*, the A- class homeotic genes *APETALA1* (*AP1*) and *APETALA2* (*AP2*) specify sepal identity and in combination with the B- class genes, *APETALA3* (*AP3*) and *PISTILLATA* (*PI*), specify petal identity. When B- is combined with C- class, conferred by the gene *AGAMOUS* (*AG*), stamen identity is specified, whereas C- class alone specifies carpel identity and floral determinacy (Coen and Meyerowitz, 1991). Later, the ABC model was expanded and classes D and E were included. The D- class genes, *SEEDSTICK* (*STK*) and *SHATTERPROOF* (*SHP*) 1 and 2, are required for ovule identity within the carpel (Favaro et al., 2003; Pinyopich et al., 2003). The E- class genes *SEPALLATA* (*SEP*) act redundantly in the specification of sepals, petals, stamens, carpels and ovules by participating in complexes with the A, B, C, and D proteins (Pelaz et al., 2000; Ditta et al., 2004; Castillejo et al., 2005).

Previous studies of flower development genes in grapevine were based on the identification and functional analysis of *Vitis vinifera* subsp. *vinifera* homologous of the corresponding ABCDE *Arabidopsis* genes (Boss et al., 2001, 2002; Calonje et al., 2004; Sreekantan et al., 2006; Poupin et al., 2007). The grapevine A*P1* homologous gene, *VviAP1*, is expressed during flower development, becoming excluded from the sepal-forming region, being preferentially expressed in the growing petals, stamens, and carpels (Calonje et al., 2004). The A- class homeotic gene *AP2* is the only non-MADS box gene that acts as a negative regulator of *AG*, as observed in *ap2 Arabidopsis* mutant flowers in which sepals are replaced by carpels (Yant et al., 2010). In the grapevine genome there are several genes encoding AP2/ERF proteins expressed in both vegetative and reproductive tissues at different developmental stages (Licausi et al., 2010).

Several studies have shown that the expression of *VviPI* and *VviAP3* in the cultivated *Vitis* is consistent with *PI* and *AP3* expression in *A. thaliana*, being restricted to the petal and stamen whorls (Poupin et al., 2007; Díaz-Riquelme et al., 2009). In the hermaphrodite grapevine *VviTM6*, the homolog of *TOMATO MADS BOX GENE 6* (*TM6*), a gene closely related to *AP3* (Kramer and Irish, 2000), is expressed during flower organ identity in the three inner whorls as well as during fruit growth and ripening (Díaz-Riquelme et al., 2009).

In cultivated grapevine the expression pattern of the E- class *VviSEP1* and *VviSEP3* genes, is similar to the *Arabidopsis* counterparts. *VviSEP1* is expressed in all floral whorls whereas *VviSEP3* expression is only excluded from sepal whorl (Boss et al., 2002).

The ABCDE model genes have been long considered as candidate genes for sex determination in monoecious and dioecious species. In the dioecious species *Spinacia oleracea*, with unisexual flowers (Sather et al., 2010), the expression of B- class floral identity genes is absent in female flowers whereas in male flowers the genes are strongly expressed (Pfent et al., 2005). In male plants, *SpPI-*silencing originates normal female flowers, indicating that sexual dimorphism occurs through the regulation of B- class gene expression that, by suppressing the formation of the gynoecium act as masculinizing genes (Sather et al., 2010). Contrary to *Spinacia*, in the dioecious *Silene latifolia*, unisexuality arises by organ abortion (Hardenack et al., 1994). The expression patterns of *SlM2* and *SlM3*, the *PI* and *AP3* homologous genes, differ during male and female flower organogenesis. At early flower development stages *SlM2* and *SlM3* are exclusively expressed in petal and stamen primordia, both in male and in female flower meristems (Hardenack et al., 1994). However, during the late stages of female flower development, *SlM2* is not expressed in the primordial of the aborted stamen (Kazama et al., 2005). In the dioecious *Rumex acetosa*, C- class transcripts were detected in the third and fourth whorls of young male and female flowers. However, in male flowers the expression in the carpel whorl is transient, disappearing from the arrested fourth-whorl, while in female flowers the expression is retained in the carpel but is absent from the stamen primordia (Ainsworth et al., 1995, 2005).

Another important gene acting during flower development is *SUPERMAN* (*SUP*), necessary to the establishment of the boundaries between third and fourth whorl. *SUP* is thought to coordinate the proliferation of stamen and carpel specific meristematic cells, keeping the proper structure of the whorls and maintaining the boundary between whorl 3 and whorl 4 at the right position (Sakai et al., 2000). The *SUP* homolog of *S. latifolia, SlSUPERMAN* (*SlSUP*), shows gender-specific expression. *SlSUP* is a female flower specific gene, expressed in the second and third whorls and in the ovules, suggesting that *SlSUP* has a positive role in female flower development (Kazama et al., 2009). Also in *Cucumis sativus*, a *SUP* ortholog (*CsSUP*) is predominantly expressed in female organs, and absent in male flowers (Zhao et al., 2014), suggesting a conserved role in flower organ determination.

In female and male flowers of wild *Vitis vinifera*, the involvement of homeotic genes in the establishment of dioecy or flower type specification has not yet been deeply analyzed. RNA-seq and qRT-PCR analysis during the inflorescence development of male and female plants of the dioecious species (*V. v. sylvestris*) and the hermaphrodite plants of *V. v. vinifera*, showed that there was no significant differences in the overall levels of ABCDE gene expression that could account for the specification of the different flower types (Ramos et al., 2014). However, it is possible that rather than differences in the overall level of expression of these genes, a distinct spatial pattern of expression in the three flower types during reproductive organ development could be responsible for conferring different flower organ identity. Therefore, in the current work we performed a detailed analysis of the spatial pattern of expression of the ABCDE genes during the development of male, female and hermaphrodite flowers of *Vitis*. Also, we analyzed the expression profile of *VviSUP*, to infer whether there was a different establishment of the borders between reproductive whorls in these flowers. We observed the expression profile of the genes analyzed is similar in the three flower types. Therefore, this work provides further evidence to suggest that the ABCDE genes might not be directly involved in sex specification and may act upstream of the pathways leading to organ abortion in *Vitis* unisexual flowers.

## Materials and Methods

### Plant Material

**FIGURE 1:**
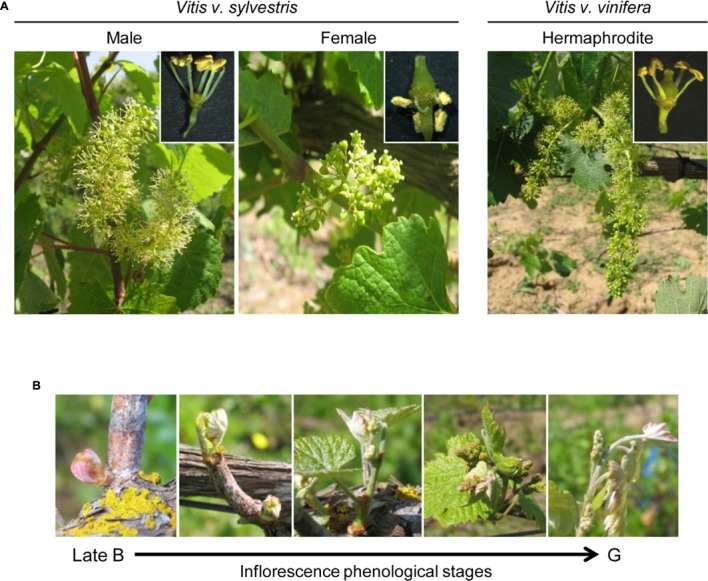
Inflorescence morphology in the three *Vitis* flower types. **(A)** Morphological disparities between the three flower types found between *V. v. sylvestris* and *V. v. vinifera*. Male plants produce a large inflorescence that display flowers with erect stamens but reduced pistil. Female plants produce clusters with few flowers bearing a complete and functional pistil but reflexed stamens with infertile pollen. Hermaphrodite plants produce a large inflorescence with both functional male and female organs. **(B)** Phenological stages of inflorescence development collected to performed *in situ* hybridization.

Inflorescences from female and male plants of *Vitis v. sylvestris* and hermaphrodite flowers of *Vitis v. vinifera* (Touriga Nacional) (**Figure 1**) were collected from the Portuguese Ampelographic Collection (PRT051), property of Instituto Nacional de Investigação Agrária e Veterinária, in Dois Portos (Lisbon district, Portugal). Inflorescence/floral buds at late stages B to G [according to phenological classification of developmental stage by Baggiolini (1952)] were collected from several male, female and hermaphrodite plants during April and May (**Figure 1**).

### RNA Extraction, cDNA Synthesis and Cloning

Total RNA was extracted from inflorescences using a plant RNA extraction kit, Spectrum^TM^ Plant Total RNA Kit (Sigma-Aldrich, Inc) following the manufacturer’s instructions. cDNA synthesis was performed by reverse transcription with heat denaturation according to the Two Step RT-PCR Procedure of RETROscript Reverse Transcription Kit (Ambion, Life Technologies, Spain). For each sample 100 ng of total RNA were used. cDNA amplification was performed through PCR in 25 μl total volume composed by 1 μg of cDNA, PCR buffer (20 mM Tris–HCl [pH 8.4], 50 mM KCl), 1.5 mM of MgCl_2_, 0.2 mM of dNTP mix, 0.4 μM of each forward and reverse primers, 5 U of Taq DNA polymerase and autoclaved MiliQ water. The initial 4 min denaturation occurred at 94 C followed by 30 cycles of 45 s at 94°C (denaturation), 45 s at 55°C (annealing), 90 s at 72°C (extension), and a final extension step of 4 min at 72 C. PCR fragments were cloned in the linearized vector pGEM^®^-T Easy Vector System (Promega, Leiden, Netherlands) according to the manufacturer’s instructions. The extraction of plasmid DNA of *Escherichia coli* cells was carried out with the PureLink^®^ Quick Plasmid Miniprep Kit (Invitrogen^TM^, Carlsbad, California) following the manufacturer’s instructions.

### Gene Sequence Identification

The protein sequence of AP1, AP2, AP3, PI, AG, SUP, SEP1, and SEP3 from *Arabidopsis* were retrieved from TAIR^3^ and TM6 from tomato was retrieved from NCBI^4^ and blasted (Camacho et al., 2009) against the *Vitis* database^5^ using the more recent annotation 12x v2.1. The same approach was used to identify the same genes in the other species.

Phylogenetic analysis of protein sequences was performed with the Maximum Likelihood method through MEGA (Molecular Evolutionary Genetics Analysis) version 6 (Tamura et al., 2013). The bootstrap consensus tree was inferred from 1,000 replicates.

### RNA *in Situ* hybridization

Plant tissue fixation, clearing and *in situ* hybridization experiments were performed as previously described (Coen et al., 1990; Coito et al., 2017). Primers for riboprobes synthesis used in the *in situ* hybridization were design using the software Primer Premier 5.0 (Premier Biosoft International) using a primer length of 20 ± 2 bp (Supplementary Table 1). cDNA probes were cloned into the pGEM^®^ T-easy vector system and amplified by PCR with the M13 forward/reverse primers and specific forward/reverse primers (Supplementary Table 1). The PCR product was purified using the MinElute PCR Purification Kit (QIAGEN, Valencia, CA, United States), according to the manufacturer’s instructions, and used as template for the riboprobe synthesis, which was carried with SP6 and T7 RNA polymerase to obtain the sense and antisense strands. The paraffin embedded material was sectioned at 7 μm and the tissue slices mounted with distilled water. Images were captured with a fluorescence microscope (Wild Leitz, Laborlux S) with an AxioCam HRM (Zeiss). Hybridizations were performed at 50°C with the exception of *VviAP3* and *VviTM6* that were performed with higher stringency at 55°C due to relatively high homology between both probes. All *in situ* hybridization procedures were made with sense (data not show) and anti-sense probes.

## Results and Discussion

The ABCDE model genes are good candidates to be involved in the establishment of male and female flowers in dioecious species, particularly the B- and C- class genes due to their role in reproductive organ identity. Therefore, it is essential to analyze the expression of these genes in a systematic way, regarding the temporal and spatial dynamics throughout flower development in male, female and hermaphrodite *Vitis* plants.

### Phylogenetic Analysis of ABCDE Model Genes in *Vitis*

Several grapevine genes involved in flower organ identity have been previously reported (Boss et al., 2001, 2002; Calonje et al., 2004; Poupin et al., 2007; Díaz-Riquelme et al., 2009). However, in the *Vitis* genome database^6^ there are more than one gene annotated as *VviAP1*, *VviAP2*, *VviAG*, *VviSEP1*, and *VviSEP3* (Supplementary Table 2). Therefore, to make sure we were analyzing the tissue expression pattern during flower development of the right ABCDE homolog, phylogenetic trees were generated (Supplementary Figure 1) with the deduced amino acid sequences for the MADS-box proteins SHP1, STK, AG, PI, AP1, AP3, FRUITFULL (FUL), TM6 and SEP, and as well as the non MADS-box protein AP2 from the following species: *Vitis vinifera* (*Vvi*), *Arabidopsis thaliana* (*At*) and other dicots plants such as *Citrus sinensis* (*Ci*), *Cucumis sativus* (*Cs*), *Antirrhinum majus* (*Am*), *Malus domestica* (*Md*), *Petunia hybrida* (*Ph*), *Populus trichocarpa* (*Pt*), *Prunus persica* (*Pp*), *Castanea mollissima* (*Cm*), *Solanum lycopersicum* (*Sl*), *Pinus radiate* (*Pr*), and the monocot plant *Orysa sativa* (*Os*) (Supplementary Table 3). The resulting phylogenetic trees (Supplementary Figure 1A) show that *VviAP1* proteins (VIT_201s0011g00100, VIT_214s0083g01030 and VIT_217s0000g04990) are grouped in three clades. In the current work, we have chosen the gene VIT*_*201s0011g00100 to work with (*VviAP1*), previously identified as the closest *AtAP1* homolog (Calonje et al., 2004). Three *VviAP2* (VIT_207s0031g00220, VIT_208s0040g03180 and VIT_213s0019g03550) genes were found in the *Vitis* genome. Nevertheless, VIT_207s0031g00220 displays higher protein homology with *AP2* genes with proven functions (Supplementary Figure 1A) and also, RNA-seq data (flowers from *V. v. sylvestris* and *V. v. vinifera*) showed that VIT_207s0031g00220 is expressed in distinct flower developmental stages (Ramos et al., 2014) suggesting that *VviAP2* could be a worthy candidate to fulfill the *AP2* canonical function.

As far as B- class genes are concerned, the two *VviAP3* genes annotated (VIT_218s0001g13460 and VIT_204s0023g02820) were grouped in the AP3 and TM6 clade, respectively (Supplementary Figure 1B). In a previous study VIT_204s0023g02820 was assigned as *VviTM6* (Poupin et al., 2007). In order to clarify these annotations, we took a closer look into the *VviAP3* genes (VIT_218s0001g13460 and VIT_204s0023g02820). The B- class gene *AP3*/*TM6*, form a divergent linage with C-terminal specific motifs. The motif DLTTFALLE define the euAP3 linage present in higher eudicot plants such as *Arabidopsis thaliana*, *Petunia hybrid*, and *Antirrhinum majus* while the motif DLRLA is present in the paleoAP3 linage in lower eudicots, dicots and monocots (Vandenbussche et al., 2003). Comparison of VIT_218s0001g13460 and VIT_204s0023g02820 protein sequences allowed the identification of the DLRLA motif in VIT_204s0023g02820, while VIT_218s0001g13460 shows the euAP3 motif: DLTFTLLE (Supplementary Figure 2). Therefore, in this study we considered VIT_218s0001g13460 gene as *VviAP3* and VIT_204s0023g02820 gene as *VviTM6* (Poupin et al., 2007). Regarding *VviPI* (VIT_218s0001g01760), also a B- class gene, falls in the PI clade (Supplementary Figure 1B).

The C- class gene, *VviAG* (VIT_210s0003g02070), and the D- class genes, *VviSHP1* (VIT_212s0142g00360) and *VviSTK* (VIT_218s0041g01880), belong to the same sub-family, as suggested by a previous work (Becker and Theissen, 2003; Pinyopich et al., 2003). *VviSHP1* (VIT_212s0142g00360) present in chromosome 12 was subsequently considered to be the *AG* homolog (Joly et al., 2004) (Supplementary Figure 1C). In the current work we decided to carry out a detailed analysis of these three protein sequences. The data show that the VviSHP1 protein sequence shares higher similarity with AtSHP2 (82%) than with AtSHP1 (70%) (Supplementary Figures 3A,B). *VviAG*, is present on chromosome 10 (VIT_210s0003g02070) was chosen, that is the one with highest homology to *AtAG*.

The genes associated with the E- class, *VviSEP1* (VIT_214s0083g01050) and *VviSEP3* (VIT_201s0010g03900) are highly related with SEPALLATA homologs of other species, (Supplementary Figure 1D) and were previously described as *VviSEP1* and *VviSEP3*, respectively (Boss et al., 2002; Joly et al., 2004).

### Expression of Floral Identity Genes During *Vitis* Flower Development

#### A-Class Genes: *VviAPETALA*1 and *VviAPETALA*2

In *Arabidopsis thaliana*, *AP1* together with *AP2*, contributes to sepal and petal identity in the first and second whorls (Theissen, 2001; Krizek and Fletcher, 2005). Defective mutants in *AP1* exhibit various defective phenotypes due to the role of this gene in organ and floral meristem identify along with *LEAFY* (Wagner et al., 1999). *ap1* flowers have sepals converted into bracts and additional flowers are formed in the axis of the bracts (Irish and Sussex, 1990) suggesting that *AP1* not only specifies the identities of sepals and petals but also determines the identity of floral meristem (Theissen and Saedler, 2001). Studies of eudicot species point to a conservation of the role of the *AP1* gene in the floral meristem specification. However, their involvement in the development of perianth organs is unclear (Litt, 2007; Rijpkema et al., 2010). Even in some *Arabidopsis ap1* mutants sepals are still formed proving the dubious role of *AP1* perianth identity (Bowman et al., 1993; Yu et al., 2004; Castillejo et al., 2005).

**FIGURE 2:**
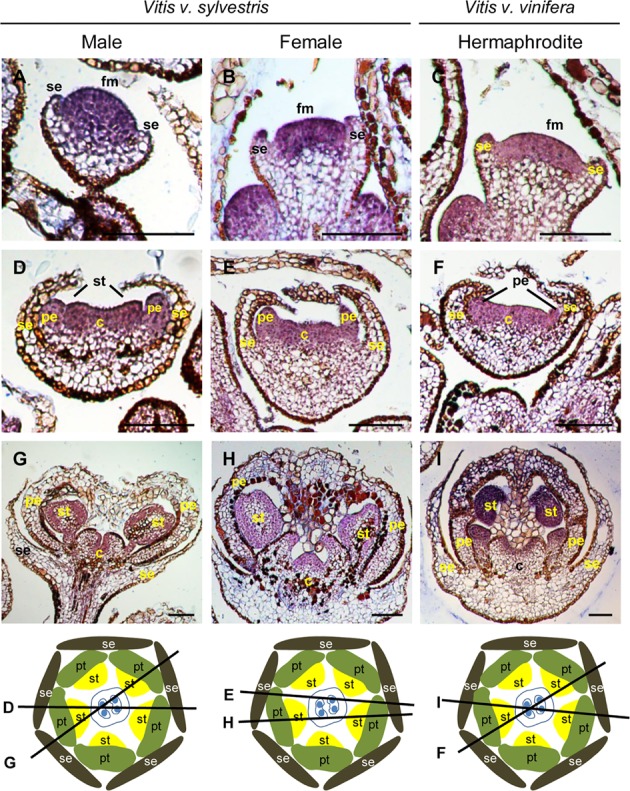
*In situ* hybridization of A- class gene *VviAPETALA1* (*VviAP1*) in developing flowers. *VviAP1* (VIT_201s0011g00100) expression analysis on longitudinal sections of wild and hermaphrodite *Vitis* inflorescences. Male: **A,D,G**; Female: **B,E,H**; Hermaphrodite (Her): **C,F,I**. The expression pattern is similar in the three flower types throughout development and is first detected in the central dome of the flower **(A–C)** when sepal formation start developing. When petals emerge, *VviAP1* remaining in the second, third, and fourth whorl **(D–F)**. At later developmental stages, *VviAP1* expression weakens at the petal base but remains in the third and fourth whorl **(G–I)**. The diagrams represent cross-sections with the corresponding figure indicated on each line. Abbreviations are as follows: fm, flower meristem; se, sepals; pe, petals; st, stamens; c, carpel. Scale bar, 100 μm.

In *Vitis*, expression of *VviAP1* occurs early in floral meristem development in the three flower types (**Figures 2A–C**), in accordance with its potential floral meristem identity role (Mandel et al., 1992). However, when stamens start to develop, *VviAP1* expression persists in the center of the flower primordium, instead of being restricted to the first and second whorls (where sepals and petals form) (**Figures 2D–F**). The *VviAP1* expression pattern is similar in the three flower types from early to later flower developmental stages and is detected in petal, stamen and carpel primordia (**Figure 2**). However, it is almost absent in petals, when they encapsulate the stamens and carpel (**Figures 2G–I**). The absence of *VviAP1* expression in the sepal regions and its expression in the flower center was not expected, considering that in *Arabidopsis* the expression of *AtAP1* is restricted to the sepal and petal whorls and absent from the developing stamens and carpels (Sundstrom et al., 2006). However, the *Vitis* expression pattern in not completely unforeseen. The expression of an *AP1* homolog in the carpel region was also reported for the *SQUAMOSA* (*SQUA*) gene in *A. majus* (Huijser et al., 1992) as well as for the *AP1-like* gene in *Gerbera hybrida* (Yu et al., 1999). Also, in grapevine it has been reported the presence of *VviAP1* transcripts in the third and fourth whorls (Calonje et al., 2004). *AP1* is canonically credited with two functions in flower development: (1) flower meristem initiation and (2) perianth identity (Huijser et al., 1992). However, variability in perianth organs identity exists and seems to be a consequence of a multiple events of evolution within angiosperms, as has been achieved through phylogenetic reconstructions using A- function genes (Zanis et al., 2003; Hileman and Irish, 2009). In the case of *Vitis* the absence of *VviAP1* expression in the flower first whorl reinforce the unclear role of this gene in sepal specification in species other then *Arabidopsis*.

**FIGURE 3:**
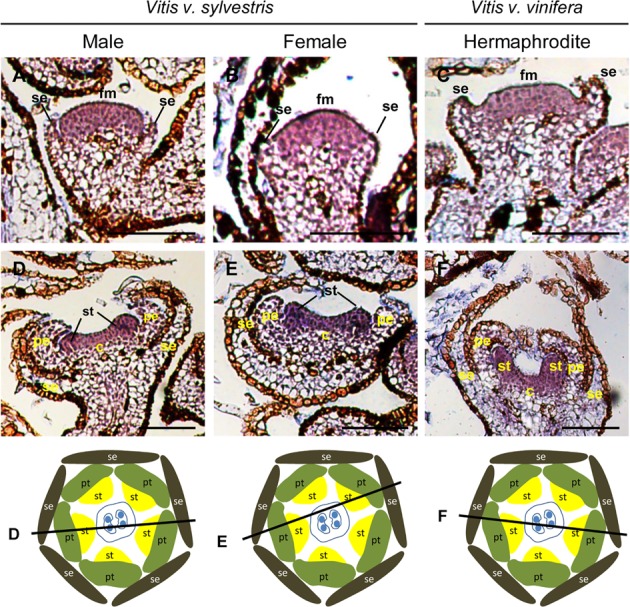
*In situ* hybridization of A- class gene *VviAPETALA2* (*VviAP2*) in developing flowers. *VviAP2* (VIT_207s0031g00220) expression analysis on longitudinal sections of wild and hermaphrodite *Vitis* inflorescences. Male: **A,D**; Female: **B,E**; Hermaphrodite (Her): **C,F**. *VviAP2* expression is similar in the three flower types throughout flower development and is first detected in the central dome of the flower meristem **(A–C)** but absent in the sepal primordia. When petals and stamens start to emerge the expression is restricted to the third and four whorls **(D–F)** where it remains. The diagrams represent cross-sections with the corresponding figure indicated on each line. Abbreviations are as follows: fm, flower meristem; se, sepals; pe, petals; st, stamens; c, carpel. Scale bar, 100 μm.

In *Arabidopsis*, *AP2* is the other A- class gene that plays a role in specifying sepals and petals (Huala and Sussex, 1992; Jofuku et al., 1994; Husbands et al., 2009). *AtAP2* is also expressed in the third and fourth whorls (Jofuku et al., 1994), where it is post-transcriptionally targeted by *miRNA172* (Chen, 2004). *ap2* flowers have leaf-like structures or carpels instead of sepals and stamens or stamenoid petals instead of petals (Bowman et al., 1989, 1991; Kunst et al., 1989). In *Vitis*, *VviAP2* expression is detected in early flower meristem and becomes excluded from early sepal primordia (**Figures 3A–C**). The expression of *VviAP2* in early flower primordia suggests a conserved role in flower meristem identity (Huala and Sussex, 1992). When petal primordia initiate, *VviAP2* is excluded from this whorl (**Figures 3D–F**) and remains in the third and fourth whorl throughout later stages of flower development. In *Arabidopsis*, *AP2* is expressed in all whorls throughout flower development (Jofuku et al., 1994; Wurschum et al., 2006; Zhao et al., 2007) but more recently, the *AP2* expression pattern was reanalyzed reporting a distinct behavior from the one previously described (Wollmann et al., 2010). These authors report that *AP2* is expressed in sepals but is absent from the center of flower primordia. Subsequently, *AtAP2* mRNA is excluded from the first whorl and appears in stamen and petal primordia. Later, *AtAP2* signal remains in petals, stamens and carpels including the ovules (Wollmann et al., 2010). The presence of *AtAP2* in the stamens and carpels shows that not only *AtAP2* has a role in ovule development (Wollmann et al., 2010) but also that *AtAG* does not antagonize *AtAP2* transcripts in the stamens (Wollmann et al., 2010). To perform this work, Wollmann et al. (2010) used a probe against the 3′ region of the transcript to avoid cross hybridization with other *AP2* homologs. In *Vitis* the full-length *VviAP2* transcript was used and it is possible that the detection of mRNA in the dome of the flower meristem is a result of cross hybridization. However, Wollmann et al. (2010) also tested the full-length cDNA of *AtAP2* and the accumulation pattern of mRNA was similar with the expression pattern using only a 3′ region RNA probe.

In *Vitis* the expression of *VviAP2* suggests (1) that this gene may not be fundamentally necessary for sepal or petal formation; (2) its transient expression during primordia initiation could be the trigger for first and second whorl identities or (3) may be part of a complex set of genes that act redundantly to establish the identity of the first and second whorl. The results obtained in *Vitis* seem to corroborate the results reported in *A. thaliana* (Krogan et al., 2012) being suggestive of a role of *VviAP2* in floral meristem identify, as its mRNA accumulation is observed early in flower development (**Figures 3A–C**) and later may be acting synergistically with B- and C- class genes in the third and fourth whorl, respectively, to specify the reproductive organs.

Although *AP1* and *AP2* show a widespread role in flower meristem initiation, the association regarding their role in organ identity has been troublesome (reviewed in Litt and Kramer, 2010). The results, regarding *Vitis AP1* and *AP2* contribute to the mystery surrounding the role of A- function genes regarding perianth identity across the core eudicots (Theissen et al., 2000; Maes et al., 2001; Shepard and Purugganan, 2002; Smyth, 2005; Morel et al., 2017) and reinforce the intricacy of these set of genes.

#### B- Class Genes: *VviAPETALA3, VviPISTILLATA*, and *VviTM6*

**FIGURE 4:**
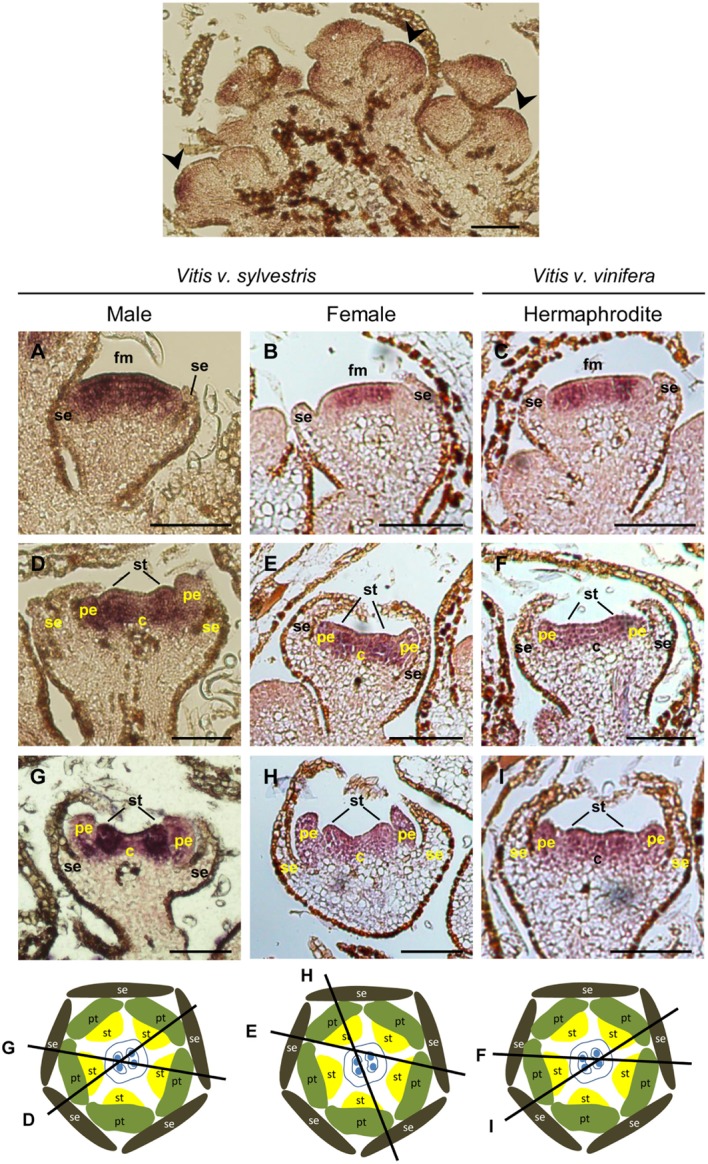
*In situ* hybridization of B- class gene *VviAPETALA3* (*VviAP3*) in developing flowers. *VviAP3* (VIT_18s0001g13460) expression analysis on longitudinal sections of wild and hermaphrodite *Vitis* inflorescences. Upper panel: in early stages of inflorescence development the expression pattern is detected in the central dome of the flower meristem (arrowhead), before sepal primordia emerge, being similar in the three flower types. Male: **A,D,G**; Female: **B,E,H**; Hermaphrodite (Her): **C,F,I**. *VviAP3* expression is similar in the three flower types throughout flower development and is first detected in the central dome of the flower meristem **(A–C)**. When petals emerge the expression starts to be restricted to the second and third whorl being excluded from sepals **(D–I)**. The diagrams represent cross sections with the corresponding figure indicated on each line. Abbreviations are as follows: fm, flower meristem; se, sepals; pe, petals; st, stamens; c, carpel. Scale bar, 100 μm.

Early in development of *Arabidopsis* flowers, *PI* is expressed in the central dome of the flower meristem (Goto and Meyerowitz, 1994). Later, the expression of *AP3*/*PI* is detected in the second and third whorls where the petals and stamens are specified (Bowman et al., 1989). *TM6*, considered a B-class homeotic gene (Poupin et al., 2007), was first identified in tomato and is expressed in stamens and in carpels (Pnueli et al., 1994; de Martino et al., 2006). *AP3*/*PI* are responsible for petal (along with *AP2*) and stamen (together with *AG*) identity. Mutations in B- class genes lead to sepaloid structures formed in the second whorl and carpeloid structures in the third whorl (Hill and Lord, 1989; Jack et al., 1992; Wuest et al., 2012). In *Arabidopsis*, the *AP3* gene is expressed only after sepal identity has been established and is confined to the second and third whorl. Even in early stages of development, the *AP3* mRNA is not detected in the central region of the flower meristem (Goto and Meyerowitz, 1994). In grapevine, *in situ* hybridization showed that *VviAP3* is expressed in the carpel whorl in the three flower types. In early stages of *Vitis* flower development, before sepal primordia start to emerge, high levels of *VviAP3* were detected in the central region of the flower meristem in the cells that will contribute to the formation of petal, stamen and carpel primordia (**Figure 4**, Upper panel). When sepal primordia become visible, *VviAP3* is still detected in the central dome of the flower primordia (**Figures 4A–C**). After the emergence of the petal primordia, *VviAP3* expression is present in the second and third whorls and its absence from the carpel whorl is inconclusive (**Figures 4D–F**). This pattern is common to the three flower types (**Figures 4G–I**). However, previous work, using RT-qPCR, showed that the expression of *VvAP3* is restricted to petals and stamens in a hermaphrodite variety of *Vitis v. vinifera* (Poupin et al., 2007). Due to the sequence similarity between *VviAP3* and *VviTM6* (Supplementary Figure 2) and in order to avoid cross hybridization with *VviTM6*, it was necessary to increase the stringency of *VviAP3* probe hybridization (see Material and Methods). Nevertheless, we do not rule out the possibility that some signal in the hybridization with the *VviAP3* probe may have the contribution from the *VviTM6* RNA. In all hybridizations with higher stringency the results were consistent and there is a strong possibility that *VviAP3* transcript does not accumulate in the carpel whorl (**Figure 4**).

**FIGURE 5:**
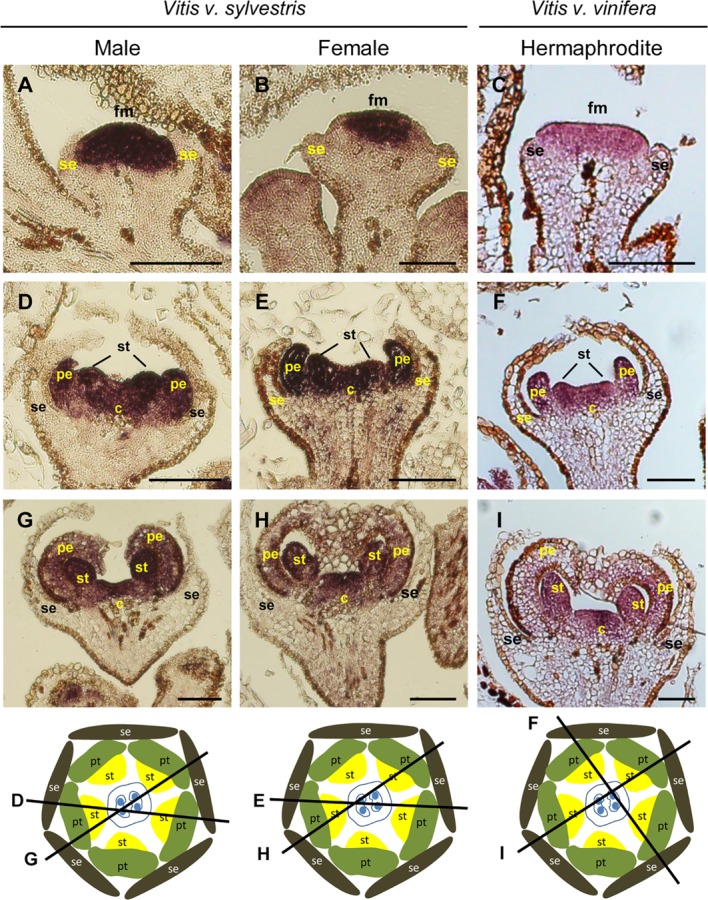
*In situ* hybridization of B- class gene *VviTM6* in developing flowers. *VviTM6* (VIT_04s0023g02820) expression analysis on longitudinal sections of wild and hermaphrodite *Vitis* inflorescences. Male: **A,D,G**; Female: **B,E,H**; Hermaphrodite (Her): **C,F,I**. *VviTM6* expression is similar in the three flower types throughout flower development and is first detected in the central dome of the flower **(A–C)**. When petals emerge *VviTM6* expression is restricted to the second, third and fourth whorls **(D–F)** where it remains **(G–I)**. The diagrams represent cross sections with the corresponding figure indicated on each line. Abbreviations are as follows: fm, flower meristem; se, sepals; pe, petals; st, stamens; c, carpel. Scale bar, 100 μm.

*TM6* is considered a B- class homeotic gene, and was first identified as being expressed in petals, stamens and carpels of *L. lycopersicum* flowers (Pnueli et al., 1994; de Martino et al., 2006). In *Vitis*, in early stages of female, male, and hermaphrodite grapevine flower primordia with already emerging sepals, the highest levels of *VviTM6* expression was detected in the center of the flower meristem (**Figures 5A–C**), being excluded from the sepal whorl (**Figures 5A–C**). After the development of petal and stamen primordia (**Figures 5D–F**), *VviTM6* was confined to the second, third and fourth whorls in the three flower types. *VviTM6* seems equally expressed in all three flower types when stamen primordia start emerging (**Figures 5G–I**), which does not suggest a preferential role in stamen development as seen by the analysis of the *TM6* function in tomato (de Martino et al., 2006). As reported in tomato (de Martino et al., 2006), the gene silencing of *TM6* by RNAi generates flowers with a compromised stamen development, however, no change in *VviTM6* expression was observed between functional stamens of male and reflexed stamens of female flowers in *Vitis* suggesting that *VviTM6* is not involved in stamen abortion. In the Petunia *ap3* mutant (that lacks petals and stamens) is complemented with 35S-driven *PhTM6*, petal development is restored (Rijpkema et al., 2006), suggesting a role in petal development. In grapevine, the presence of *VviTM6* mRNA in the three inner whorls points to its participation in the development of petals, stamens, and carpels.

**FIGURE 6:**
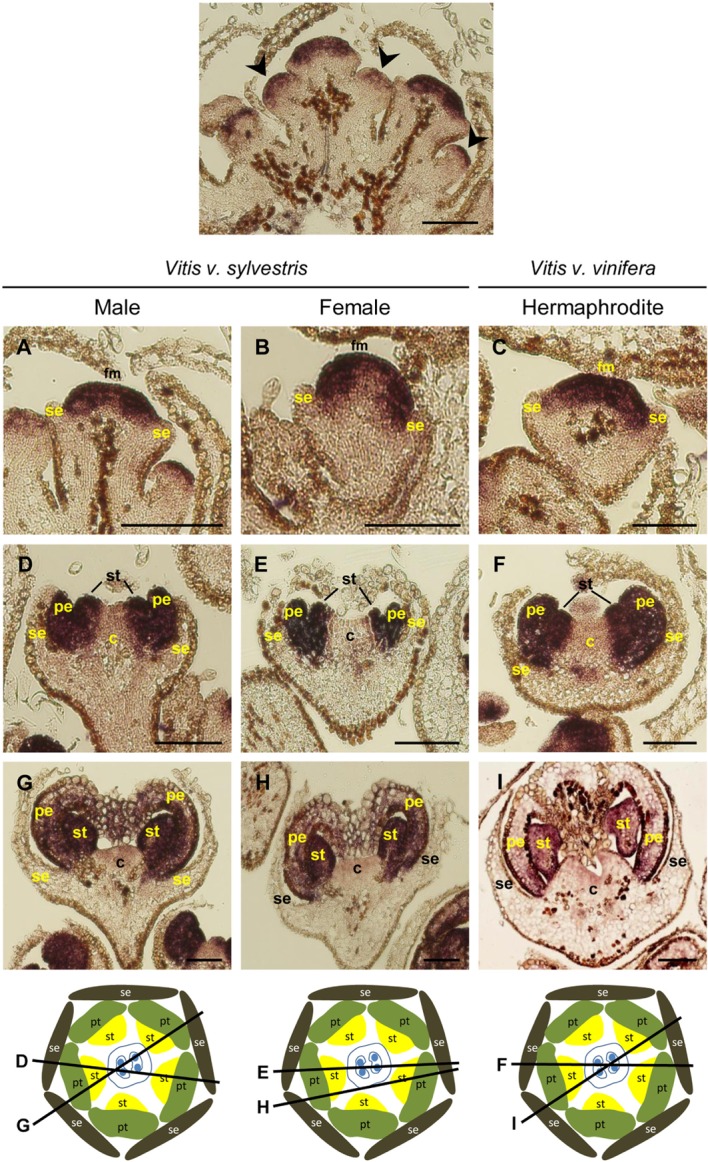
*In situ* hybridization of B- class gene *VviPISTILLATA* (*VviPI*) in developing flowers. *VviPI* (VIT_218s0001g01760) expression analysis on longitudinal sections of wild and hermaphrodite *Vitis* inflorescences. Upper pane: in early stages of inflorescence development the expression is detected in the central dome of the flower meristem (arrowheads), before sepal primordia emerge and is similar in the three flower types. Male: **A,D,G**; Female: **B,E,H**; Hermaphrodite (Her): **C,F,I**. *VviPI* expression is similar in the three flower types throughout flower development and is first detected in the central dome of the flower meristem **(A–C)**. When petals start to emerge *VviPI* is excluded from the first and fourth whorl, but is present in the second and third whorl **(D–F)** where it remains **(G–I).** The diagrams represent cross sections with the corresponding figure indicated on each line. Abbreviations are as follows: fm, flower meristem; se, sepals; pe, petals; st, stamens; c, carpel. Scale bar, 100 μm.

*PISTILLATA* in *Arabidopsis* is expressed in cells that will give rise to petals, stamens, and carpel primordia in early stages of flower development (Hill and Lord, 1989; Sundstrom et al., 2006), and its expression is confined to the second and third whorls only in later stages (Goto and Meyerowitz, 1994). In early stages of *Vitis* flower development, *VviPI* is expressed in the center of the flower meristem (**Figure 6**, Upper panel) similar to what was described for *Arabidopsis* (Goto and Meyerowitz, 1994). When sepal primordia start to emerge, *VviPI* has a high expression in the cells that will develop into petals and stamens and starts to fade from the region that will develop into carpels (**Figures 6A–C**). As soon as petal primordia starts to emerge, *VviPI* is completely excluded from the fourth whorl (**Figures 6D–F**) remaining confined to the second and third whorl during the later stages of development (**Figures 6G–I**). *VviPI* seems to be more expressed in stamen than in petal primordia, both in male and in female flowers. This expression pattern is similar in female and male flowers and did not follow the pattern of organ abortion founded in *Silene lafolia*, where at later stages of female flower development *PI* expression is absent from the aborted stamens (Kazama et al., 2005).

Together, the expression of *VviPI* and *VviAP3* B- class genes is similar to what was described for the B- class *Arabidopsis* homologs (Goto and Meyerowitz, 1994). The early dynamics of both *VviAP3* and *VviPI* (**Figures 4**, **6**) raises questions regarding the molecular mechanism underlying the activation of both genes in the floral meristem in *Vitis*. Our results suggest that the boundaries of *VviAP3* and *VviPI* are identical during the onset of *Vitis* flower meristem suggesting, at least in part, that both genes could be under the regulation of identical upstream factors.

#### The Cadrastral Gene: *VviSUPERMAN*

**FIGURE 7:**
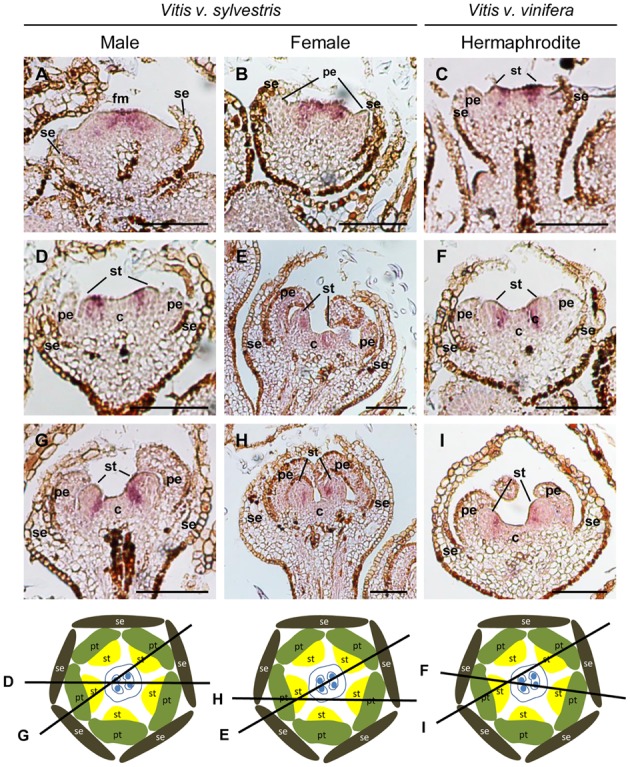
*In situ* hybridization of *VviSUPERMAN* (*VviSUP*) in developing flowers. *VviSUP* (VIT_205s0049g00070) expression analysis on longitudinal sections of wild and hermaphrodite *Vitis* inflorescences. Male: **A,D,G**; Female: **B,E,H**; Hermaphrodite (Her): **C,F,I**. *VviSUP* expression is similar in all three flower types throughout flower development. *VviSUP* is detected in early stages of flower development in the central area of the flower meristem in cells that will define the boundary between third and fourth whorl **(A–C)**. When stamens emerge, *VviSUP* is well settled out in the cells that divide those two whorls **(D–F)** where it remains at least until sepals fuse and enclose the flower bud **(G–I)**. The diagrams represent cross sections with the corresponding figure indicated on each line. Abbreviations are as follows: fm, flower meristem; se, sepals; pe, petals; st, stamens; c, carpel. Scale bar, 100 μm.

The *SUP* gene establishes the boundaries between stamen and carpel whorls by acting as a cadastral factor restricting the B- class genes expression (Hiratsu et al., 2002). In *sup Arabidopsis* mutants, the expression of *AP3*/*PI* expands to the fourth whorl where, staminoid structures are formed instead of a carpel (Bowman et al., 1991, 1992). *SUP* acts by inhibiting the expression of *AP3* and *PI* in the fourth whorl of the developing flower (Jack et al., 1992; Sakai et al., 2000). In *Vitis* inflorescences, the expression of *VviSUPERMAN* (*VviSUP*) is similar during flower development of the three flower types (**Figure 7**). The earliest expression of *VviSUP* is detected in the central region of the floral meristem adjacent to the boundary between the third and fourth whorls (**Figures 7A–C**). In later stages of floral development, *VviSUP* is expressed in the inner region of the developing stamen primordia (**Figures 7D–F**). When stamen primordia are clearly defined, *VviSUP* expression remains in the region adjacent to the carpel primordia (**Figures 7G–I**).

The *in situ* results obtained with *VviAP3*/*VviPI* and their regulator *VviSUP* show that *VviAP3* expression appears first in the central region of flower meristem and is, in a later stage, followed by *VviSUP* expression, which goes in agreement with what has been observed in *Arabidopsis* mutants, in which *SUP* expression is detect after the initiation of *AP3* expression (Sakai et al., 1995).

Our data suggest that early *VviSUP* expression act in a similar way to what has been described in *Arabidopsis*, maintaining the whorl 3 and 4 boundary after the whorl prepattern has been established. In *Arabidopsis*, *SUP* gene appears to act transiently, only required for a short period before the cells in the floral whorls undergo extensive divisions to produce organ primordia (Sakai et al., 2000), a brief expression that is sufficient to fulfill *SUP* function in whorl boundary maintenance (Bowman et al., 1991, 1992; Jack et al., 1992). However, in grapevine, the expression of *VviSUP* persists in the inner part of whorl 3 until later flower developmental stages (**Figures 7G–I**) suggesting that it may not be the trigger but could be involved in maintain the genetic signaling by controlling the balanced proliferation of two adjacent floral whorls.

#### C- Class Genes: *VviAGAMOUS*

**FIGURE 8:**
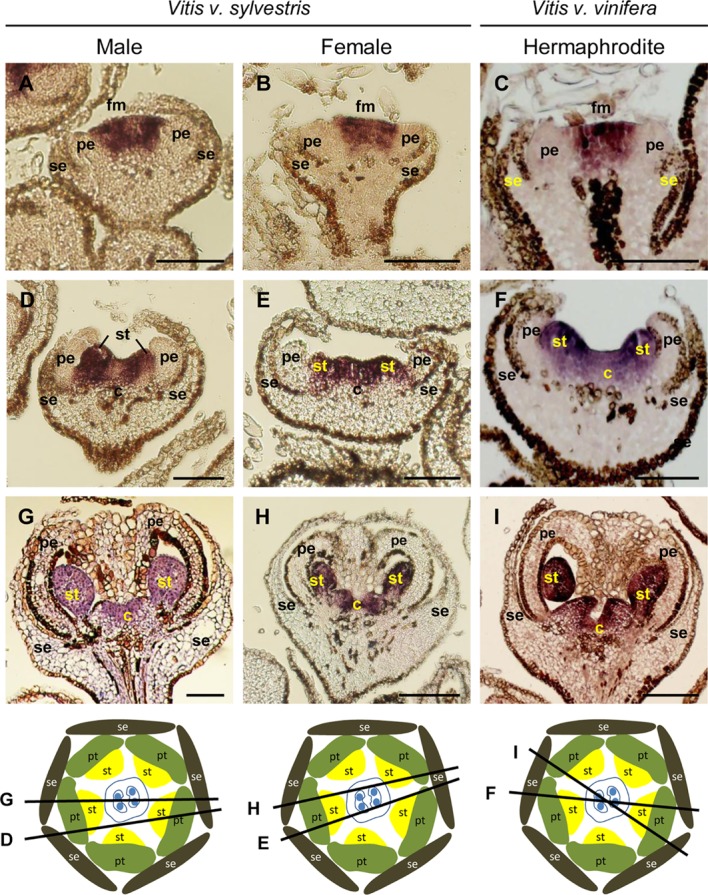
*In situ* hybridization of C- class gene *VviAGAMOUS* (*VviAG*) in developing flowers. *VviAG* (VIT_210s0003g02070) expression analysis on longitudinal sections of wild and hermaphrodite *Vitis* inflorescences. Male: **A,D,G**; Female: **B,E,H**; Hermaphrodite (Her): **C,F,I**. The location of the *VviAG* RNA is similar throughout flower development in the three flower types. *VviAG* is first detected in the central dome of the flower **(A–C)**. When petal primordia emerge **(D–F)** *VviAG* is detected in the third and fourth whorls where it remains throughout flower development **(G–I)**. The diagrams represent cross sections with the corresponding figure indicated on each line. Abbreviations are as follows: fm, flower meristem; se, sepals; pe, petals; st, stamens; c, carpel. Scale bar, 100 μm.

*AGAMOUS* is a C- class homeotic MADS-box gene expressed both in the third whorl, acting together with *PI* and *AP3* in specifying stamen identity, and in the fourth whorl, where it is responsible for carpel identify and, to some degree, ovule identity (Bowman et al., 1989, 1991). Mutant plants for *AG* do not have reproductive organs, and instead exhibit a phenotype described as “a flower within a flower” with the absence of stamens and carpels (Yanofsky et al., 1990). In *Vitis*, *VviAG* expression (VIT_210s0003g02070) follows a temporal and spatial pattern (identical in all the three flower types) that fits with what was proposed by the ABCDE model. *VviAG* is expressed in the center of the *Vitis* flower meristem (**Figures 8A–C**) and is confined to the third and fourth whorls when stamen primordia emerge (**Figures 8D–F**). This expression pattern is maintained throughout later stages of flower development (**Figures 8G–I**). A similar expression profile has been reported in the hermaphrodite Riesling variety, in which *VviSHP1* is expressed in later stages of flower development (Joly et al., 2004) where it is required to control ovule identity on tissues that develop within the carpels (Pinyopich et al., 2003). In the dioecious *Rumex acetosa*, *AG* transcript accumulation decrease in the aborted organs (Ainsworth et al., 1995), however, in grapevine the similar expression profile of *VviAG* in all flower types (Ramos et al., 2014) may exclude the direct involvement of *VviAGAMOUS* in carpel abortion or the formation of reflexed stamens.

#### E- Class Genes: *VviSEPALLATA*1 and *VviSEPALLATA*3

**FIGURE 9:**
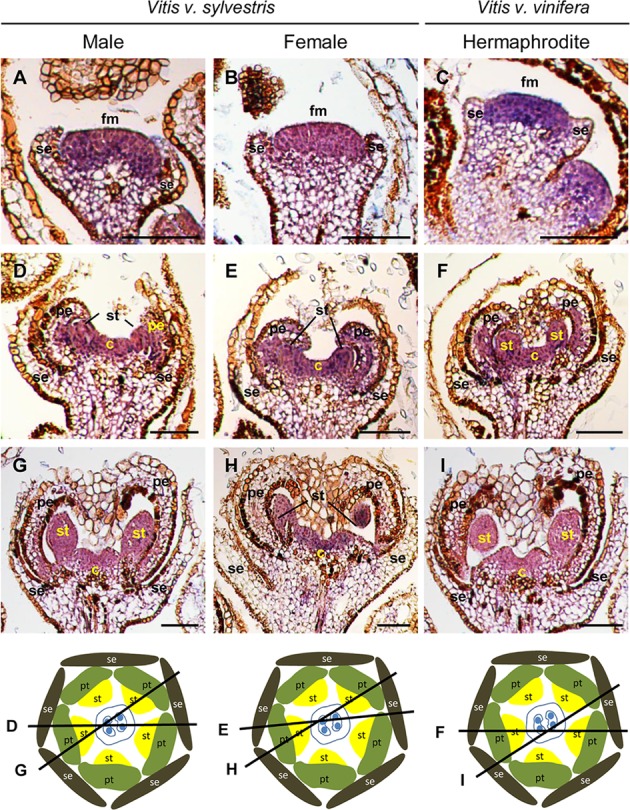
*In situ* hybridization of E- class gene *VviSEPALLATA1* (*VviSEP1*) in developing flowers. *VviSEP1* (VIT_214s0083g01050) expression analysis on longitudinal sections of wild and hermaphrodite *Vitis* inflorescences. Male: **A,D,G**; Female: **B,E,H**; Hermaphrodite (Her): **C,F,I**. *VviSEP1* expression is similar in the three flower types throughout flower development and is first detected in the central dome of the flower **(A–C)**. When petals start to develop the expression is restricted to the second, third and four whorls **(D–F)** where it remains throughout flower development but with lower expression in the second whorl **(G–I)**. The diagrams represent cross sections with the corresponding figure indicated on each line. Abbreviations are as follows: fm, flower meristem; se, sepals; pe, petals; st, stamens; c, carpel. Scale bar, 100 μm.

The E- class *SEPALLATA* (*SEP*) play a central role in flower meristem determinacy and organ identity (Ditta et al., 2004). The *SEP* genes, although functionally redundant, are required for the formation of petals, stamens and carpels as the triple mutant (*sep 1/2/3*) has an indeterminate flower with all flower organs converted into sepals (Pelaz et al., 2000; Honma and Goto, 2001). In *A. thaliana*, *SEP1* is expressed throughout flower development (Savidge et al., 1995). In the three *Vitis* flower types, *VviSEP1* is expressed in the center of the flower meristem (**Figures 9A–C**), but excluded from the sepal primordia. When petal primordia emerge, *VviSEP1* remains in the second, third and fourth whorls (**Figures 9D–F**) and at later developmental stages, *VviSEP1* is still expressed in the third and fourth whorls but has lower expression in the petals (**Figures 9G–I**).

**FIGURE 10:**
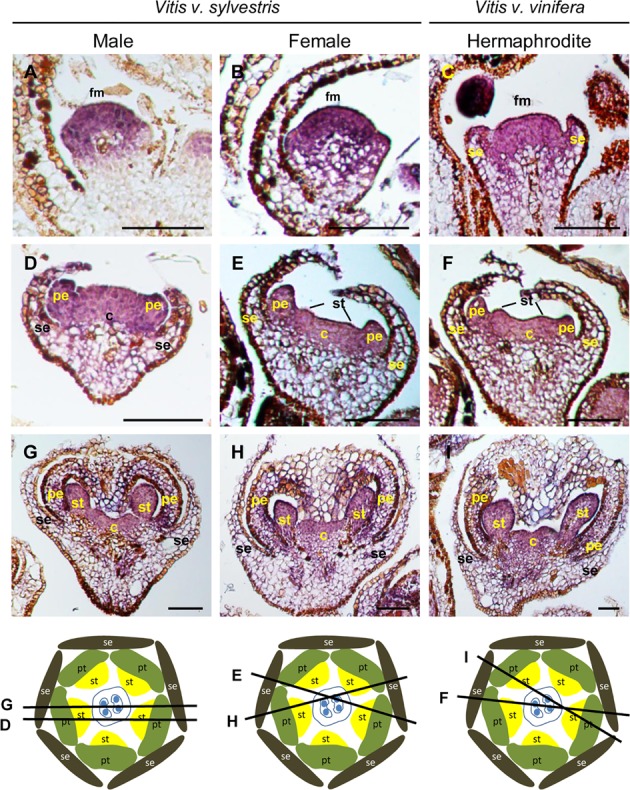
*In situ* hybridization of E- class gene *VviSEPALLATA3* (*VviSEP3*) in developing flowers. *VviSEP3* (VIT_01s0010g03900) expression analysis on longitudinal sections of wild and hermaphrodite *Vitis* inflorescences. Male: **A,D,G**; Female: **B,E,H**; Hermaphrodite (Her): **C,F,I**. *VviSEP3* expression is similar in the three flower types throughout flower development. *VviSUP3* is first detected in the central dome of the flower **(A–C)**. When petals emerge *VviSEP3* expression is excluded from the first whorl **(D–F)**. In later development stages *VviSEP3* remains in the second, third and fourth whorls **(G–I)**. The diagrams represent cross sections with the corresponding figure indicated on each line. Abbreviations are as follows: fm, flower meristem; se, sepals; pe, petals; st, stamens; c, carpel. Scale bar, 100 μm.

*SEP3* is involved in sepal, petal, stamen, carpel, and ovule development and its ectopic expression is enough to activate *AtAP3* and *AtAG* (Pelaz et al., 2000; Favaro et al., 2003; Ditta et al., 2004). In *Vitis, VviSEP3* expression is detected very early in the floral meristem in all flower types (**Figures 10A–C**). When petal and stamen primordia emerge, *VviSEP3* is excluded from sepals (**Figures 10D–F**). In later stages of flower development, *VviSEP3* is weakly expressed in both the base and adaxial side of petals (**Figures 10G–I**). No differences were found in the expression of *VviSEP3* in the three flower types (**Figure 10**). In *Arabidopsis SEP1* is expressed slightly earlier than *SEP3*, which is expressed in a region corresponding to the inner three whorls just before the initiation of floral primordia (Flanagan and Ma, 1994; Savidge et al., 1995; Ditta et al., 2004). In *Vitis*, the activities of *VviSEP1/3* span the three inner whorls similar to what happens in *Arabidopsis* (Pelaz et al., 2000). However, and contrarily to what happens in *Arabidopsis*, our data show that *VviSEP1* expression is spatially and temporally very similar to *VviSEP3*.

## Conclusion

The results of the present study reinforce the great complexity of the events and molecular cascades that occur during determination and specification of floral organ identity in *Vitis*. Previous results (RNA-seq and qRT-PCR) showed similar levels of ABCDE gene expression in male, female and hermaphrodite *Vitis* flowers (Ramos et al., 2014), suggesting that these genes may not be directly involved in sex specification in this species. However, similar levels of expression can be produced by different patterns of tissue expression and, therefore, there was still a possibility that these ABCDE genes could mediate the specification of the three different *Vitis* flower types.

**FIGURE 11:**
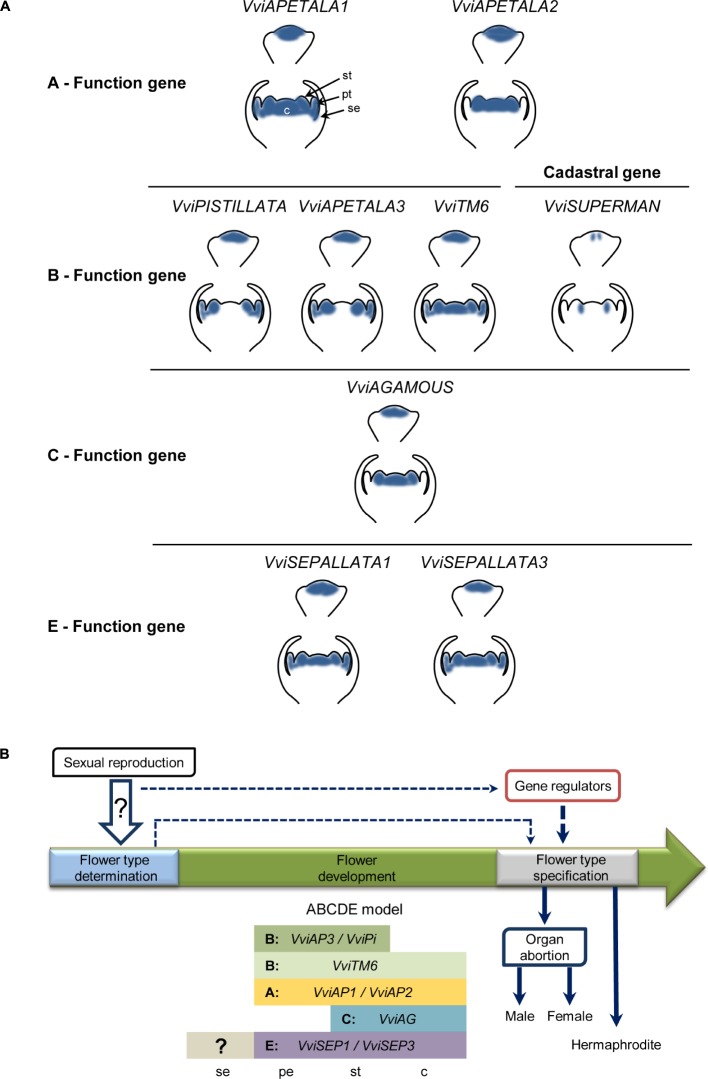
Homeotic genes expression in *Vitis* and their role in flower sex development. **(A)** Diagrams of flowering genes expression. Expression pattern of the ABCDE model genes and the cadastral gene *VviSUPERMAN*. Homeotic genes *VviAPETALA* (*VviAP1*) and *VviAP2* (*VviAP2*) from the A- function have no expression in the first whorl of sepals. They are expressed in petals, stamens and carpel primordia. The genes *VviAPETALA* (*VviAP3*) and *VviPISTILATA* (*VviPI*) from the B- function form petals and stamens in the second and third whorl. *VviSUPERMAN* restricts B- function expression genes in flower meristems in different flower developmental stages of *V. v. vinifera* and a *V. v. sylvestris. VviAGAMOUS* (*VviAG)* is the only gene in the C- function responsible for carpel formation in the fourth whorl. Our data suggest that there are no antagonism between A- and C- function in *Vitis*. E- function *VviSEPALLATA* (*VviSEP*) genes act redundantly and are expressed in the three inner whorls and required for the correct formation of all flower organs. **(B)** Proposed model for flower development. The model suggests that flower determination is established at the moment of reproduction by yet unknown factors. The flower development is regulated through the onset of the ABCDE model genes until a later stage of development, as observed in this work. Downstream of homeotic genes that establish flower organ identity and initiation, other regulators might be involved in flower type specification. These regulators can either (I) act under the control of the initial unknown sexual determination factors or (II) the initial unknown sexual determination factors are directly involved in flower specification into male and female. Both scenarios can be exclusively or act together promoting organ abortion in *V. v. sylvestris* or a hermaphrodite flower development in *V.v. vinifera*. se, sepals; pe, petals; st, stamens; c, carpel.

Despite some slight differences, the homeotic genes exhibit a spatial expression profile similar in the three flower types and analogous to what has been described for *Arabidopsis* homologs. Additionally, none of these genes fall into the region of chromosome 2 responsible for sex determination (Fechter et al., 2014; Picq et al., 2014; Coito et al., 2017; Zhou et al., 2017). Moreover, our results show that despite these genes being involved in flower organ identity they are not directly responsible for flower type specification in *Vitis.* One outcome that emerges from this work concerns the identity of sepals (**Figure 11A**). The homeotic genes, *VviAP1* and *VviAP2*, have no expression in the first whorl where they are supposed to regulate the formation of sepals (**Figure 11A**). This unexpected absence of expression raises two hypotheses: these genes may not be involved in sepal identity specification in this species or the structure known as sepals has, in fact, an identity different from canonical sepals. Additionally, their expression in flower development at initial stages also point to a role in flower meristem identity.

A model for flower development, shown in **Figure 11B**, proposes that flower determination is established at the time of fertilization by factors still unknown (**Figure 11B**) present in autosomal chromosomes. The subsequent flower organ development may be regulated through the onset of the ABCDE homeotic genes that act after sex determination and upstream of flower organ abortion regulators, such as the already studied gene *VviAPRT3* (Coito et al., 2017). These putative regulators can act under the control of the initial unknown sexual determination factors or each of the later may be directly controlling the flower specification promoting the abnormal development of male or female reproductive organs in later stages of flower development.

Both scenarios can be exclusive or act together leading to organ abortion as in *V. v. sylvestris* flowers or hermaphrodite flowers as in *V. v. vinifera*.

## Author Contributions

MR, JLC, HS, and MMRC conceived and designed the experiments. JLC, HS, MM, and MJNR performed the experiments. HS, JLC, MJNR, MMRC, and MR analyzed the data. HS, JLC, MJNR, SA, MMRC, and MR wrote the paper. JC, JLC, and MR established *Vitis vinifera sylvestris* collection and collected plant tissues according phenological developmental stage.

## Conflict of Interest Statement

The authors declare that the research was conducted in the absence of any commercial or financial relationships that could be construed as a potential conflict of interest.

## Footnotes

1 http://www.genoscope.cns.fr/externe/GenomeBrowser/Vitis/entry_ggb.html

2 http://genomes.cribi.unipd.it/grape/

3 https://www.arabidopsis.org/

4 https://www.ncbi.nlm.nih.gov/

5 http://genomes.cribi.unipd.it/gb2/gbrowse/public/vitis_vinifera_v2/

6 http://genomes.cribi.unipd.it/grape/
