# Supplementary material for: Vitis Flower Sex Specification Acts Downstream and Independently of the ABCDE Model Genes
Source: Front Plant Sci. 2018 Jul 16;9:1029. doi: 10.3389/fpls.2018.01029 (PMC6055017; doi:10.3389/fpls.2018.01029)
Supplement: Supplementary file 1 [file Presentation_1.PDF]

Supplementary Material

Vitis flower sex specification acts downstream and independently of the ABCDE model genes

João Lucas Coito[1§], Helena Silva[2§], Miguel Jesus Nunes Ramos[1], Miguel Montez[1], Jorge Cunha[3], Sara Amâncio[1], Maria Manuela Ribeiro Costa[2], Margarida Rocheta[1*]

[1]Linking Landscape, Environment, Agriculture and Food (LEAF), School of Agriculture, University of Lisbon, Tapada da Ajuda, 1359-017 Lisboa, Portugal

[2]Biosystems and Integrative Sciences Institute (BioISI), Plant Functional Biology Centre, University of Minho, Campus de Gualtar, 4710-057 Braga, Portugal

[3]Instituto Nacional de Investigação Agrária e Veterinária, Quinta d'Almoinha, Dois Portos, Portugal.

*** Corresponding Author:**
rocheta@isa.ulisboa.pt

§ Both authors contributed equally to this work

Supplementary Figures and Tables:

(A)

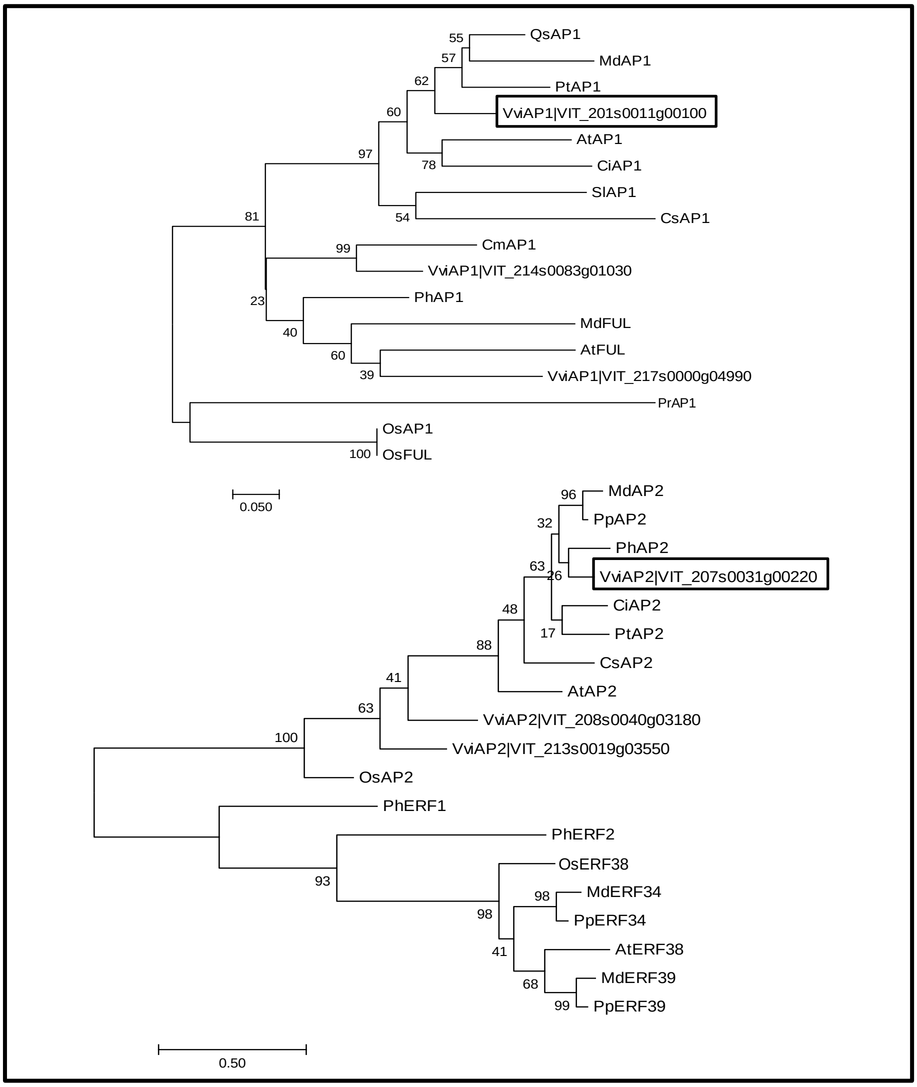

(B)

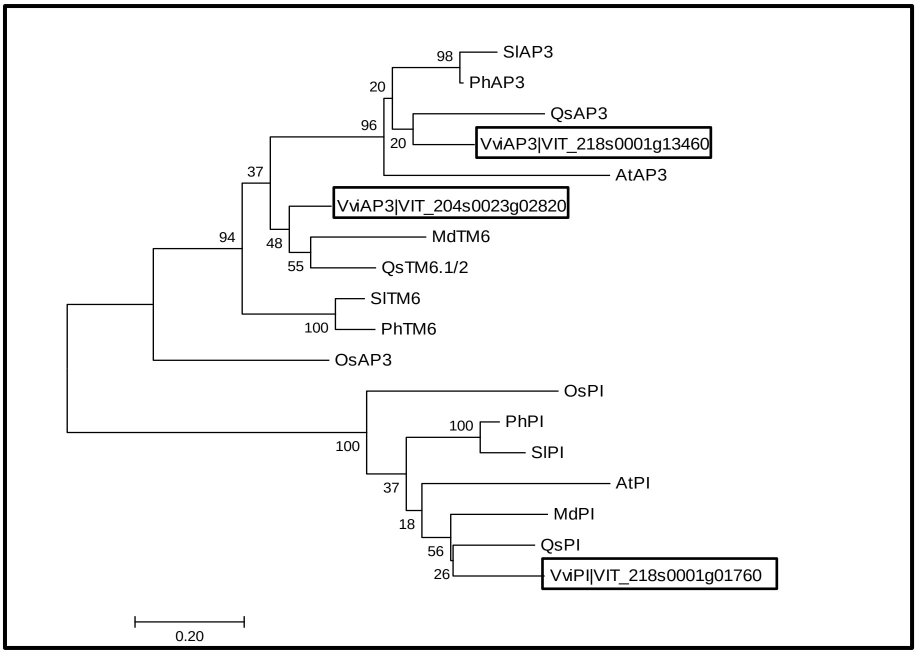

(C)

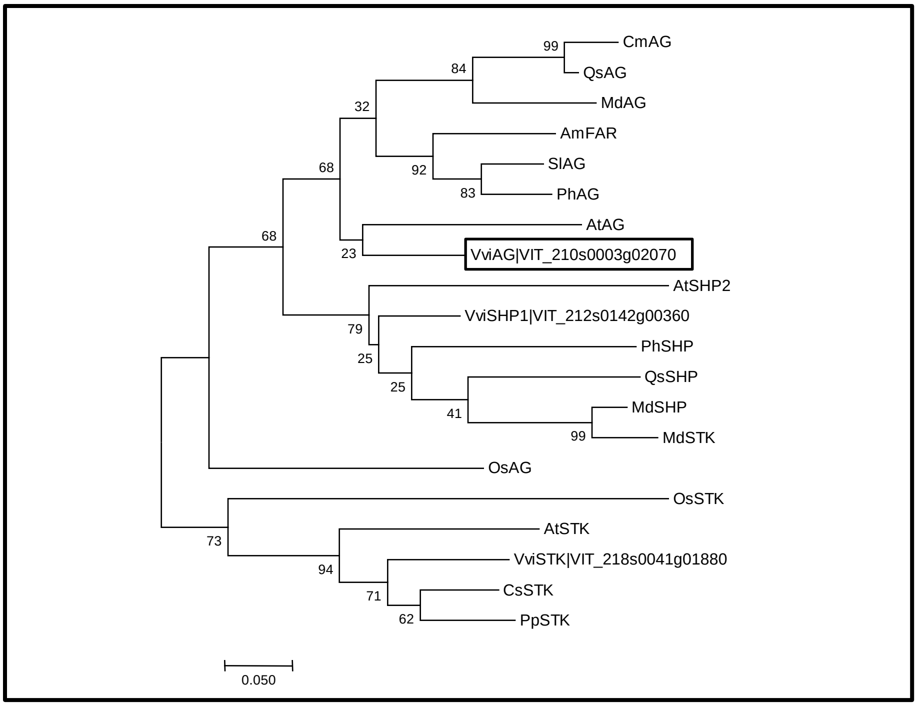

(D)

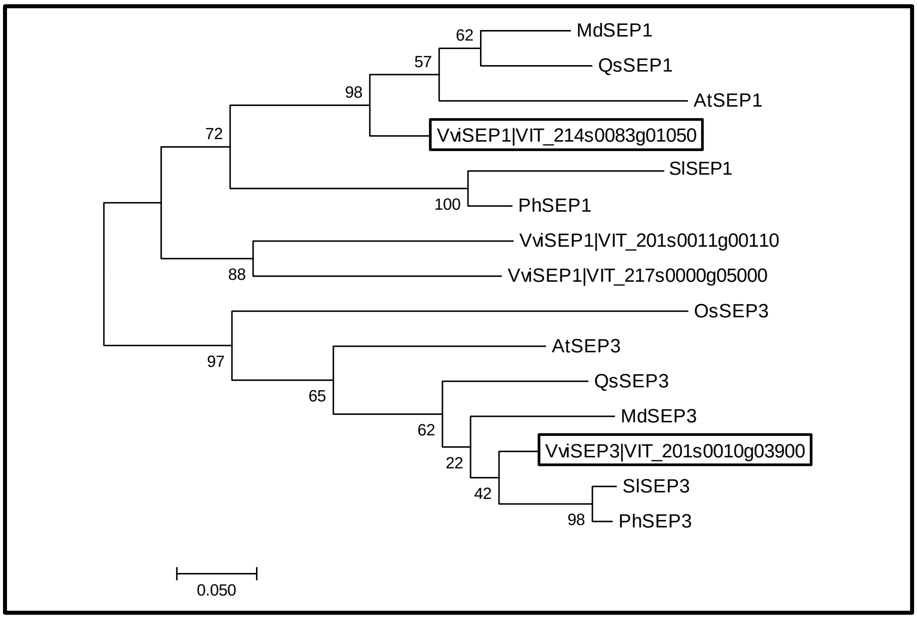

Supplementary Figure 1. Phylogenetic analysis of flower homeotic genes from different plant species. *Vitis vinifera vinifera* (*Vvi*) ABCDE amino acid sequences were deduced using the cDNA sequence of gene ID from CRIBI (http://genomes.cribi.unipd.it/grape/) and translated using the ExPASy translate tool (http://web.expasy.org/translate/). Phylogenetic analysis of homeotic genes protein sequences was performed with the Maximum Likelihood method through MEGA (Molecular Evolutionary Genetics Analysis) version 6. The bootstrap consensus tree was inferred from 1000 replicates and represents the evolutionary history of the protein analyzed. (A) APETALA1 (AP1) and APETALA2 (AP2); (B) APETALA3 (AP3), TOMATO MADSBOX6 (TM6) and PISTILLATA (PI)*;* (C) AGAMOUS (AG), SHATTERPROOF (SHP) and SEEDSTICK (SKT); (D) SEPALLATA 1 and 3.

Am - *Antirrhinum majus*; At – *Arabidopsis thaliana*; Ci – *Citrus sinensis*; Cm – *Castanea mollissima*; Cs – *Cucumis sativus*; Md – *Malus domestica*; Os – *Oryza sativa;* Ph – *Petunia hybrida*; Pt – *Populus trichocarpa*; Pp – *Prunus persica; Pr – Pinus radiata Qs – Quercus suber*; Sl – *Solanum lycopersicum*; Vvi – *Vitis vinifera vinifera*. Accession numbers for amino acids sequences are presented in Supplementary Table 2 and 3.

VviAP3|VIT_218s0001g13460
MARGKIEIKRIENSTNRQVTYSKRRNGIFKKASELTVLCDAKVSIIMLSSTGKLHEYISPSTTTKQIFDQYQ
NTLGVDLWSYHYERMQENLKKLKDVNKNLRKEIRQRMGEHLSDLSVEELRDLEQEMESSLKMVRDRKY
QVINNQIETFKKKVRNVEQIHKNLLHEFDARDRDQHYGLVDNGGDYESVLGFSNGSSPVFALSLQPNPP
NDLHSGVGSDLTFTLLE

VviAP3|VIT_204s0023g02820
MCCLKSKDPTPHVITLIPKEPFHGFGIQHSQLHYNLTFLATYPIQKLIFLFPSSSSSSSTYCALEPHYLILEK
KEGSKARKMGRGKIEIKRIENPTNRQVTYSKRRNGIFKKAQELTVLCDAKVSLIMFSNTGKFHEYTSPTIT
TKKVYDQYQKTLGIDLWSSHYERMQENLRKLKEINNKLRREIRQRMGEDLGDLSIEDLRGLEQKMDASL
GLVRERKYHVIKTQTETYRKKVRNLEEQHGNLLLNFEAKCDDPHYGLVENDGDYESAVAFANGASNLY
AFRLHQAHPNLHHDGGYGSHDLRLA

```
VviAP3|VIT_218s0001g13460   ------------------------------------------------------------
VviAP3|VIT_204s0023g02820   MCCLKSKDPTPHVITLIPKEPFHGFGIQHSQLHYNLTFLATYPIQKLIFLFPSSSSSSST

VviAP3|VIT_218s0001g13460   ---------------------MA RGKIEIKRIENST NRQVTYSKRRNGIFKKASELTV
VviAP3|VIT_204s0023g02820    YCALEPHYLILEKKEGSKARKMGRGKIEIKRIENPTNRQVTYSKRRNGIFKKAQELTV
                                                 * . * * ********* * * ** * *  ** * ** * * * * *** ** *  . * * **

VviAP3|VIT_218s0001g13460   LCDAKVSI IMLSSTGK LHEYI SPSTTTKQIF DQYQNTLGVDLWSYHYERMQENLKKLK
VviAP3|VIT_204s0023g02820   LCDAKVSLIMFSNTGKFHEYTSPT ITTKKVYDQYQKTLG I DLWSSHYERMQENLRKLK
                            * ** * * * *:**: * . * * * :* * *   **:  ** * : : :* * *  * :* * * : * * * *   * * * * *  * * **:* * *

VviAP3|VIT_218s0001g13460   DVNKNLRKEIRQRMGEHLS DLSVEELRDLEQEMESSLKMVRDRKYQVINNQIETFKKK
VviAP3|VIT_204s0023g02820   E INNKLRRE IRQRMGEDLGDLS IEDLRGLEQKMDASLGLVRER KYHVIKTQTETYRKK
                            : :*: :* *: ****** * * . * . * * *:*:* * . * * *: * :: * *   :* *:* * *: **: . *  * *: :* *

VviAP3|VIT_218s0001g13460   VRNVEQI HKNLLHEFDARDRDQHYGLV DNGGDYESVLGFSNGSSPVFALSLQPNP
VviAP3|VIT_204s0023g02820   VRNLEEQHGNLLLNFEAKCDDPH YG LVENDGDYESAVAFANGASNLYAFRLHQAH
                            * * *:*:   *   * * *  :*: *:      *   * * * * *  :*. * * * * * . : .*: **:*   : :*:  * :

VviAP3|VIT_218s0001g13460   PNDLHSGVGSDL TFTLLE
VviAP3|VIT_204s0023g02820   PNLHHDGGYGSHDLRLA-
                            * *     * .*     . .      :  *
```

Supplementary Figure 2. Protein sequence and alignment of VIT_204s0023g02820 and VIT_218s0001g13460. Protein sequence and alignment of the *VviAP3* genes according to Expasy translation tool (http://web.expasy.org/translate/) using the cDNA sequence from CRIBI (http://genomes.cribi.unipd.it/grape/) for both genes annotated as *VviAP3*. It is possible to observe that the *VviAP3* with the ID VIT_218s0001g13460 display the C-terminal sequence DLTFTLLE while the *VviAP3* with the ID VIT_204s0023g02820 display the C-terminal sequence DLRLA corresponding to the euAP3 and paleoAP3 linage, respectively.

(A)

```
VviSHP1    --------------MGRGKIEIKRIENTTNRQVTFCKRRNGLLKKAYELSVLCDAEVAL
AtSHP1    MEEGGSSHDAESSKKLGRGKIEIKRIENTTNRQVTFCKRRNGLLKKAYELSVLCDAEVAL
                         :********************************************

VviSHP1   IVFSSRGRLYEYANNS-------------------------VRTTIERYKKVCSDSSNTG
AtSHP1    VIFSTRGRLYEYANNSFIYLLLEKKKKKKKKKNLWIYSSHVVRGTIERYKKACSDAVNPP
          ::**:***********                         ** *******.***: *

VviSHP1   SVSEANAQFYQQEASKLRRQIRDIQNLNRHILGEALSSLNFKELKNLETRLEKGISRIRS
AtSHP1    SVTEANTQYYQQEASKLRRQIRDIQNSNRHIVGESLGSLNFKELKNLEGRLEKGISRVRS
          **:***:*:***************** ****:**:*.*********** ********:**

VviSHP1   KKNELLFAEIEYMQKREIELQNSNLFLRAQIAENERAQQQ---MNLMPGSDYLSTLFI—
AtSHP1    KKNELLVAEIEYMQKREMELQHNNMYLRAKIAEGARLNPDQQESSVIQGTTVYESGVSSH
          ******.**********:***:.*::***:***. * : :    .:: *: .: .

VviSHP1   ------SKNLFFLNLKN----------------
AtSHP1    DQSQHYNRNYIPVNLLEPNQQFSGQDQPPLQLV
                .:* : :** :
```

(B)

```
VviSHP1   ---------------MGRGKIEIKRIENTTNRQVTFCKRRNGLLKKAYELSVLCDAEVAL
AtSHP2    MEGGASNEVAESSKKIGRGKIEIKRIENTTNRQVTFCKRRNGLLKKAYELSVLCDAEVAL
                         :********************************************

VviSHP1   IVFSSRGRLYEYANNSVRTTIERYKKVCSDSSNTGSVSEANAQFYQQEASKLRRQIRDIQ
AtSHP2    VIFSTRGRLYEYANNSVRGTIERYKKACSDAVNPPTITEANTQYYQQEASKLRRQIRDIQ
          ::**:************* *******.***: *  :::***:*:****************

VviSHP1   NLNRHILGEALSSLNFKELKNLETRLEKGISRIRSKKNELLFAEIEYMQKREIELQNSNL
AtSHP2    NLNRHILGESLGSLNFKELKNLESRLEKGISRVRSKKHEMLVAEIEYMQKREIELQNDNM
          *********:*.***********:********:****:*:*.***************.*:

VviSHP1   FLRAQIAENERAQQQMNLM--PGSDYLSTLFIS-------KNLFFLNLKN----------
AtSHP2    YLRSKITERTGLQQQESSVIHQGTVYESGVTSSHQSGQYNRNYIAVNLLEPNQNSSNQDQ
          :**::*:*.   *** . :   *: * * :  *       :* : :** :

VviSHP1   ------
AtSHP2    PPLQLV
```

Supplementary Figure 3. Protein sequence alignment of *SHATTERPROOF* genes from *Vitis* (*Vvi*) and *Arabidopsis* (*At*). (A) The alignment between *VviSHP1* (VIT_212s0142g00360) and *AtSHP1* revealed an amino acid identity of 70%. (B) The alignment between *VviSHP1* and *AtSHP2* revealed an amino acid identity of 82%. The *VviSHP1* is closer to *AtSHP2* than to *AtSHP1*.

Supplementary Table 1. Time of probe hydrolysis at 60° C according to the fragment size. F, forward primer; R, reverse primer. The template length includes the sequence from the T7 or SP6 binding site to the polylinker.

Gene ID	Probe	Primer sequence (5'-3')	Template length (bp)	Time of hydrolysis (min)
VIT_201s0011g00100	*VviAP1*	F- AAAGATGGGAAGAGGTAGGGTT<br>R- AGTGCCTTTGGCTTCTTTGTA	448	18
VIT_207s0031g00220	*VviAP2*	F- GTCGGAAATGGGGACTACTT<br>R- ATTGTTCTGCTTGGATGCGG	724	20
VIT_218s0001g13460	*VviAP3*	F- CTTTGTGATGCTAAGGTTTC<br>R- ACGATGGTGGGATTCAGTTG	817	12
VIT_218s0001g01760	*VviPI*	F- AAGCAACAGGCAGGTGAC<br>R- ATAAGTTTTGAGGGTAATGG	823	80
VIT_204s0023g02820	*VviTM6*	F- CACCGTTCTTTGTGATGCTAA<br>R- GAACCAGACAATAGAG	905	84
VIT_210s0003g02070	*VviAG*	F- TCCGTTTCTGAAGCCAA<br>R- ATCTGATTCTACTTATGCCT	278	-
VIT_205s0049g00070	*VviSUP*	F- GCAACAACCACAAGGTCAAG<br>R- GGAACCATCCACACCCCATT	514	5
VIT_214s0083g01050	*VviSEP1*	F- GGGAAAGTGGTGAGCAGAGTA<br>R- CCATTGACATTTTGGGCATTA	381	-
VIT_201s0010g03900	*VviSEP3*	F- TATCCACAAGGGAGGCCCTG<br>R- GCAGGCCCCAGTGTGAATAAC	571	14

Supplementary Table 2. Accession numbers for *Vitis* gene names. Abbreviations are the following: *AP1*, *APETALA1*; *AP2*, *APETALA2*; *AP3*, *APETALA3*; *PI*, *PISTILLATA*; *AG*, *AGAMOUS*; *FUL*, *FRUITFULL*; *SHP*, *SHATTERPROOF*; *SKT*, *SEEDSTICK*; *SEP*, *SEPALLATA*; *TM6*, *TOMATO MADSBOX6*.

***Vitis* species**	**Gene (ID)**
VviAPETALA1	VIT_217s0000g04990
	VIT_214s0083g01030
	VIT_201s0011g00100
VviAPETALA2	VIT_207s0031g00220
	VIT_213s0019g03550
	VIT_208s0040g03180
VviAPETALA3	VIT_218s0001g13460
VviPISTILLATA	VIT_218s0001g01760
VviTM6	VIT_204s0023g02820
VviAGAMOUS	VIT_210s0003g02070
VviSHATTERPROOF	VIT_212s0142g00360
VviSEEDSTICK	VIT_218s0041g01880
VviSEPALLATA1	VIT_201s0011g00110
	VIT_214s0083g01050
	VIT_217s0000g05000
VviSEPALLATA3	VIT_201s0010g03900,
	VIT_214s0068g01800

Supplementary Table 3. Accession numbers for non *Vitis* species amino acids sequences used for phylogenetic analysis. Abbreviations of gene names: *AP1*, *APETALA1*; *AP2*, *APETALA2*; *AP3*, *APETALA3*; *PI*, *PISTILLATA*; *AG*, *AGAMOUS*; *FUL*, *FRUITFULL*; *SHP*, *SHATTERPROOF*; *SKT*, *SEEDSTICK*; *SEP*, *SEPALLATA*; *TM6*, *TOMATO MADSBOX6*.

Species	Gene	Acession number (protein)
Arabidopsis	*AtAP1*	NP_177074.1
	AtFUL	Q38876.1
	AtAP2	P47927.1
	AtERF38	NP_181113.1
	AtAP3	NP_191002.1
	AtPI	NP_197524.1
	AtAG	NP_567569.3
	AtSHP2	AAU82079.1
	AtSTK	NP_001078364.1
	AtSEP1	NP_001119230.1
	AtSEP3	NP_564214.2
Cucumis sativus	*CsAP1*	XP_011650331.1
	CsAP2	XP_004149686.1
	CsSTK	NP_001267506.1
Malus domestica	*MdAP1*	ABG85297.1
	MdFUL	XP_008374663.1
	MdAP2	NP_001280879.1
	MdERF34	XP_008347085.1
	MdERF39	XP_008369428.1
	MdTM6	XP_008346633.1
	MdPI	NP_001280926.1
	MdAG	NP_001315863.1
	MdSHP	NP_001280918.1
	MdSTK	NP_001280758.1
	MdSEP1	AAC25922.1
	MdSEP3	NP_001280756.1
Oryza sativa	*OsAP1*	XP_015631033
	OsFUL	XP_015631034.1
	OsAP2	Q10BG6
	OsERF38	XP_015634439.1
	OsAP3	XP_015641661.1
	OsPI	XP_015640709.1
	OsAG	XP_015632490.1
	OsSTK	XP_015621182.1
	OsSEP3	XP_015648762.1

Supplementary Table 3 (cont). Accession numbers for non *Vitis* species amino acids sequences. Abbreviations of gene names: *AP1*, *APETALA1*; *AP2*, *APETALA2*; *AP3*, *APETALA3*; *PI*, *PISTILLATA*; *AG*, *AGAMOUS*; *FUL*, *FRUITFULL*; *SHP*, *SHATTERPROOF*; *SKT*, *SEEDSTICK*; *SEP*, *SEPALLATA*; *TM6*, *TOMATO MADSBOX6*.

Species	Gene	Acession number (protein)
Petunia hybrida	*PhAP1*	AAF19721.1
	PhAP2	Q9XHD4
	PhERF2	ADP37417.1
	PhERF1	ADP37416.1
	PhTM6	AAF73933.1
	PhAP3	Q07472.1
	PhPI	CAA50549.1
	PhAG	Q40885.1
	PhSHP	CAA48635.1
	PhSEP1	AAQ72498.1
	PhSEP3	Q03489.2
Prunus persica	*PpAP2*	AEB92231.1
	PpERF39	XP_007209502.1
	PpERF34	XP_007218799.2
	PpSTK	ABQ85556.1
Populus trichocarpa	*PtAP1*	AAT39554.1
	PtAP2	POPTR_0007s10780.1
Solanum lycopersicum	*SlAP1*	NP_001234665.1
	SlTM6	NP_001311309.1
	SlAP3	NP_001234077.2
	SlPI	ABG73411.1
	SlAG	NP_001266181.1
	SlSEP1	AAM33104.2
	SlSEP3	NP_001234384.1
Citrus sinensis	*CiAP1*	NP_001275828.1
	CiAP2	XP_006494466.1
Quercus suber	*QsAP1*	XP_023910685.1
	QsTM6.1/2	XP_023911257.1
	QsAP3	XP_023924416.1
	QsPI	XP_023887186.1
	QsAG	XP_023894634.1
	QsSHP	XP_023917960.1
	QsSEP1	XP_023912244.1
	QsSEP3	XP_023899435.1
Castanea mollissima	*CmAP1*	AAZ77749.1
	CmAG	AAZ77747.1
Pinus radiata	*PrAP1*	AAD09207.1
Antirrhinum majus	*AmFAR*	CAB42988.1
